# NDRindex: a method for the quality assessment of single-cell RNA-Seq preprocessing data

**DOI:** 10.1186/s12859-020-03883-x

**Published:** 2020-12-16

**Authors:** Ruiyu Xiao, Guoshan Lu, Wanqian Guo, Shuilin Jin

**Affiliations:** 1 School of Computer Science and Technology, Harbin Institute of Technology, Zhejiang, China; 2 State Key Laboratory of Urban Water Resource and Environment, Harbin Institute of Technology, Harbin, China; 3 School of Mathematics, Harbin Institute of Technology, Harbin, China

**Keywords:** Single-cell, RNA-seq, Normalization, Dimension reduction, Preprocess path

## Abstract

**Background:**

Single-cell RNA sequencing can be used to fairly determine cell types, which is beneficial to the medical field, especially the many recent studies on COVID-19. Generally, single-cell RNA data analysis pipelines include data normalization, size reduction, and unsupervised clustering. However, different normalization and size reduction methods will significantly affect the results of clustering and cell type enrichment analysis. Choices of preprocessing paths is crucial in scRNA-Seq data mining, because a proper preprocessing path can extract more important information from complex raw data and lead to more accurate clustering results.

**Results:**

We proposed a method called NDRindex (Normalization and Dimensionality Reduction index) to evaluate data quality of outcomes of normalization and dimensionality reduction methods. The method includes a function to calculate the degree of data aggregation, which is the key to measuring data quality before clustering. For the five single-cell RNA sequence datasets we tested, the results proved the efficacy and accuracy of our index.

**Conclusions:**

This method we introduce focuses on filling the blanks in the selection of preprocessing paths, and the result proves its effectiveness and accuracy. Our research provides useful indicators for the evaluation of RNA-Seq data.

## Background

Nowadays, single-cell RNA sequencing is being generally used in biology and iatrology related areas. The efficient methods used in COVID-19 researches these days would be a good example. Many researchers used single cell RNA sequencing data to determine the sensitivity of organs other than the lungs, and found that the heart, esophagus, kidney, and ileum are also munitive organs [1–4]. One of the main advantages of single-cell RNA sequencing (scRNA-Seq) is that it can be clustered unsupervised to determine cell types [5]. Normalization and dimension reduction methods are typically used for data preprocessing before clustering procedure. The normalization methods are designed to eliminate technical noise in scRNA-Seq data. Previously, many advanced normalization methods were proposed to preprocess scRNA-Seq data, such as TMM [6], SAMstrt [7], Scran [8], BASiCS [9], SCnorm [10] Linnorm [11], ORNA [12] and FSQN [13]. SAMstrt, Scran, SCnorm, Linnorm and TMM preprocesses data by calculating the scaling factor of the gene expression of each cell.

Most single-cell RNA-seq data is sparse, and almost 90% data is zero measurements. so we use dimension reduction methods to convert the high-dimensional data into low-dimensional data. Sammon [14] mapping and T-SNE [15] are dimension reduction methods that keeps the data manifold unchanged, while principal component analysis (PCA) are designed to extract the important information. Methods like LSPCA [16] and ESPCA [17] combines traditional PCA with other algorithms to overcome the shortcomings of PCA. In addition, some clustering methods also provide normalization and dimensionality reduction methods, such as Seurat [18] and SC3 [5].

Various normalization and dimension reduction methods use different data processing algorithms and obtain different clustering results. Ideally, normalization and dimension reduction methods should produce high-quality data, and the aggregation results should be meaningful. Due to poor clustering trends, completely random data is not conducive to clustering [19]. In order to solve this problem, we propose NDRindex (Normalization and Dimensionality Reduction index) to evaluate the degree of data aggregation. By comparing all combinations of normalization and dimension reduction methods, the data with highest NDRindex will be the selected for further clustering.

## Implementation

As input, NDRindex requires a gene expression matrix, normalization methods and dimension reduction methods. To make this step easier, f NDRindex includes five normalization methods TMM, Linnorm, Scale, Scarn, Seurat and three-dimensional reduction methods PCA, tSNE and Sammon.

Then NDRindex evaluates the data qualities. The prepossessed data with the highest NDRindex score are chose and saved, then outputted.

**Fig. 1:**
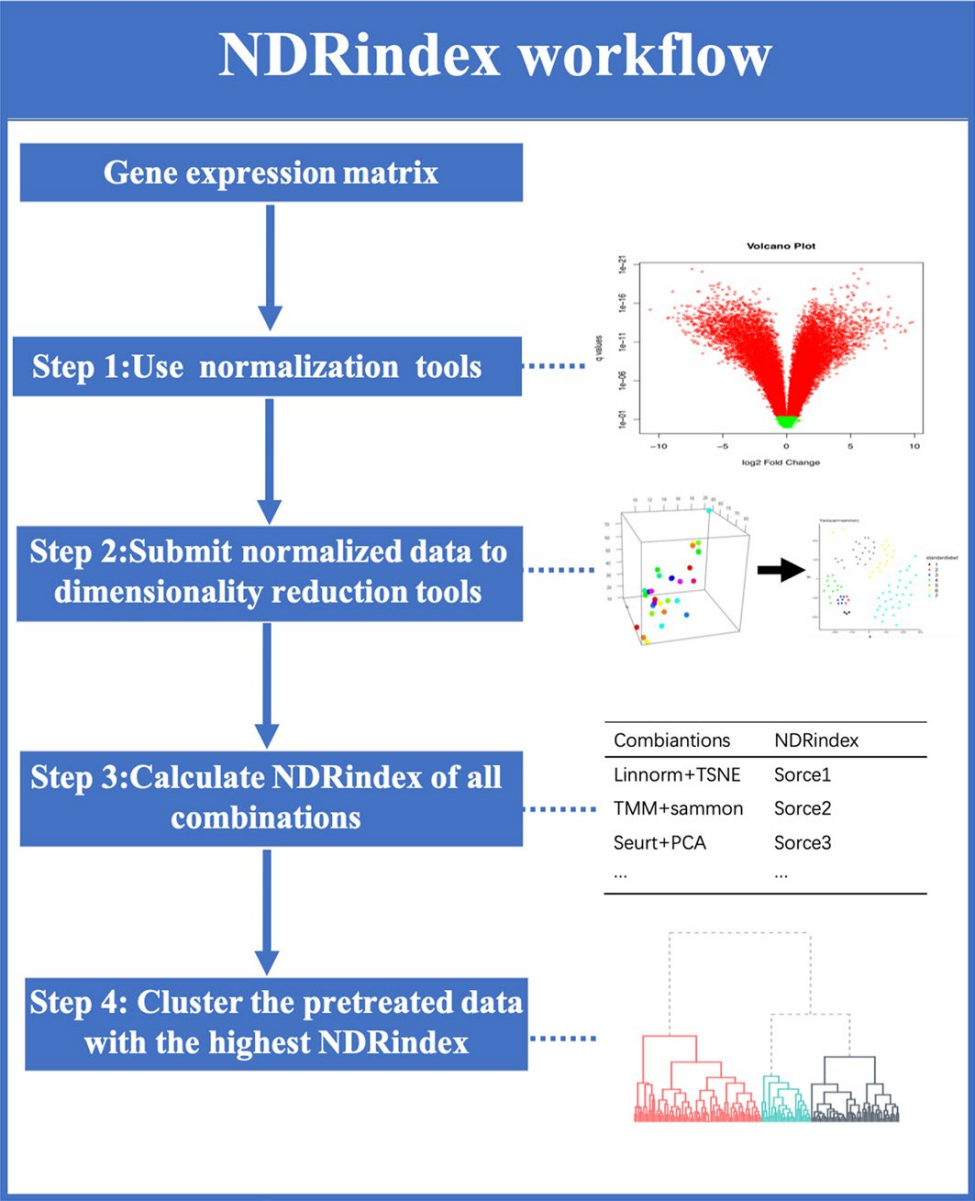
Workflow of NDRindex. First, gene expression matrix, normalization methods

Finally, clustering techniques (k-menas, hclust, etc.), are applied to the selected data. After that, the clustering result is output. The entire workflow can be described as shown in Fig. 1.

The key to the NDRindex method is an algorithm for evaluating data quality. Not all data is suitable for clustering. If the data set does not contain natural clusters, the clustering results will be meaningless, so it is very important to analyze the tendency of data clustering and evaluate its quality [19]. If the data set does not contain natural clusters, the clustering results will be meaningless, so it is very important to analyze the tendency of data clustering and evaluate its quality [19]. NDRindex algorithm evaluates the cluster tendency by calculating the aggregation degree of data. The higher the degree of clustering, the more points are distributed in a relatively small area, indicating the existence of natural clusters. However, assessing the degree of aggregation is a difficult problem. For example, given two points with the distance 50 cm. If we consider points less than 5 cm apart aggregative, the two points will be considered as two clusters. If we consider points less than 500 cm apart, the two points will probably be considered as one cluster. Thus the degree of aggregation is closely related to the distances of the points and the definition of aggregation. Based on the above assumptions, the NDR index is designed as follows:

Step 1. Calculate the distance matrix and ‘average scale’ of data.

According to experience, if the data spread over a larger area, the definition of ‘aggregative’ should be loosened; if there are more data points, the definition of ‘aggregative’ should be enforced, so it is assumed that the range of data distribution is proportional to the definition of ‘close’, and the number of data points is Inversely proportional to the definition ‘close’. The ‘average scale’ of data is defined as $\frac{M}{{\log_{10} n}}$, where M is the lower quartile distance of all point pairs and represents the range of data distribution, n is the sample number of the database. When the distance of two points is smaller than the ‘average scale’, they would be considered ‘close’.

Step 2. Clustering and find the point gathering areas.

NDRindex find the point gathering areas by the following step:

- Select a point A randomly. Let A as an individual cluster and let cluster number $K = 1$.
- Find the point B closest to geometric center of the cluster that A belongs to, if the distance between geometric center and B is smaller than average scale (defined in step1), than add B to the cluster of A and update the geometric center. Otherwise, let B as a new individual cluster, and increase the cluster number K. Repeat step b until all point belongs to a cluster.

After that, NDRindex will find some clusters, each represents a point gathering area.

Step 3. Calculating the final index.

For each cluster, the average of the distances from all points to the geometric center is defined as the cluster radius. A smaller cluster radius indicates a smaller and dense point collection area and a larger degree of clustering. Therefore, we define the final index as:

$$NDRindex = 1.0 - \frac{R}{{\frac{M}{{\log_{10} n}}}}$$

where

$$R = \frac{{\mathop \sum \nolimits_{i \in set\,of\,all\,clusters} \frac{{\mathop \sum \nolimits_{p \in i} distance\left( {p,geometric\,center\,of\,i} \right)}}{size\,of\,i}}}{K}$$

To reduce randomness, NDRindex runs this algorithm 100 times and takes the average value as the final result.

**Fig. 2:**
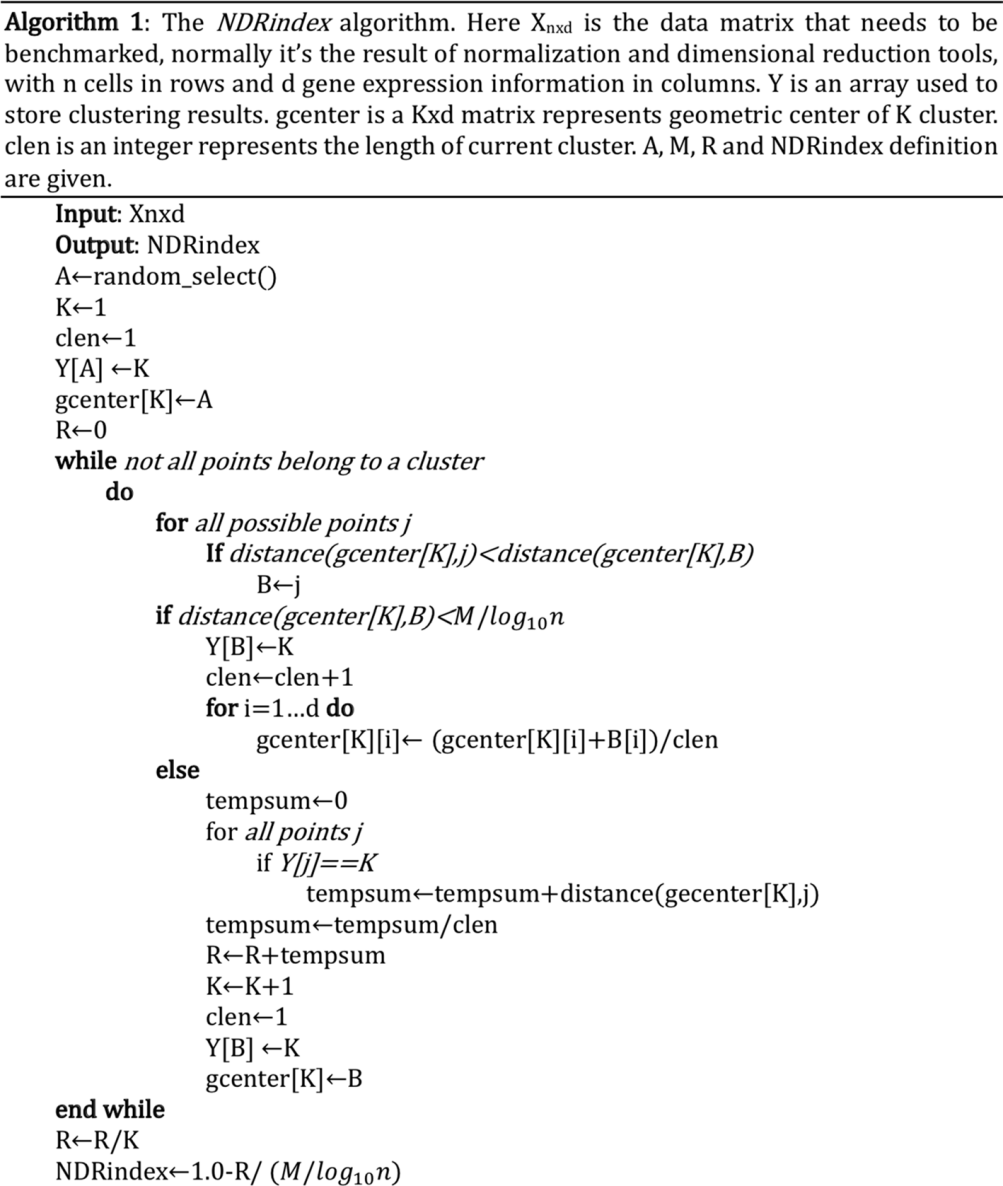
Pseudo-code of NDRindex

The procedure below can be described as pseudo-code as Fig. 2 described.

## Results

**Fig. 3:**
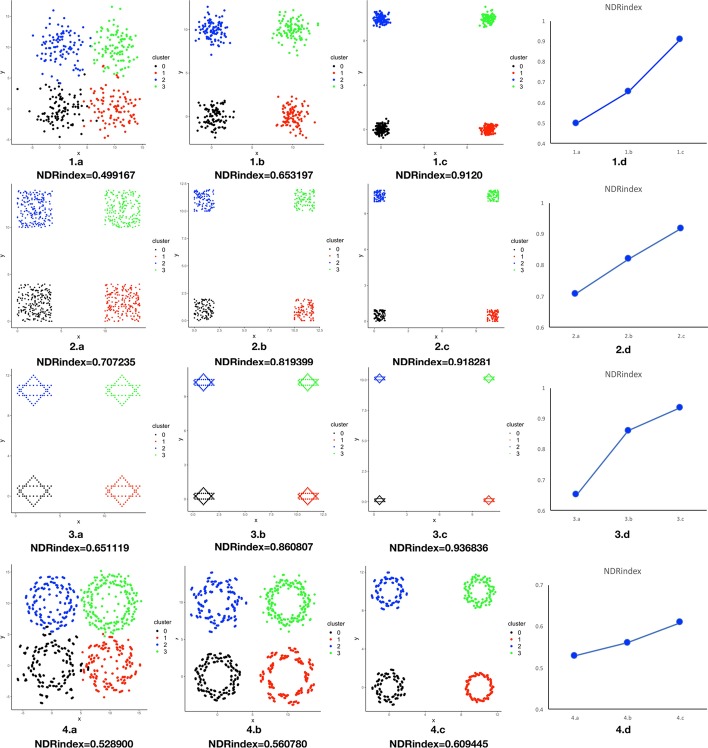
Results of NDRindex on simulative data. Every line shows one type of simulative data we test, line 1 to line 4 are two-dimensional normal distribution, square, hexagram, random shape, respectively. For each line, column a to column c are four data whose scale are decreased by order, column d is a line graph shows how NDRIndex changes with the decrease of data scale. When data become more aggregate, NDRindex always become higher

**Fig. 4:**
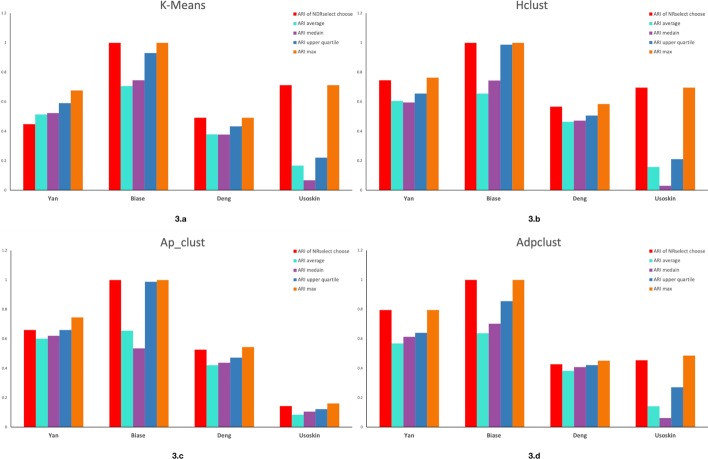
Data quality assessment of NDRindex chosen and unchosen. For each database, we test five normalization methods (TMM, Linnorm, scran, Seurat, scale) and three dimensionality reduction methods (tSNE, PCA, sammon). We select the result of each combination and submit all twelve of them to four typical clustering methods and benchmark the clustering results with ARI. Figure 3.a to 3.d shows the results of clustering methods kmeans, hclust, adpclust, ap_clust, respectively. Comparing the data NDRindex chosen (red rectangular) and the data NDRindex unchosen, we find that most of the chosen combination get the highest ARI (orange rectangular) during clustering, nearly all chosen combination get the ARI above upper quantile (blue rectangular). That means NDRindex do select high quality data that is suitable for clustering

**Fig. 5:**
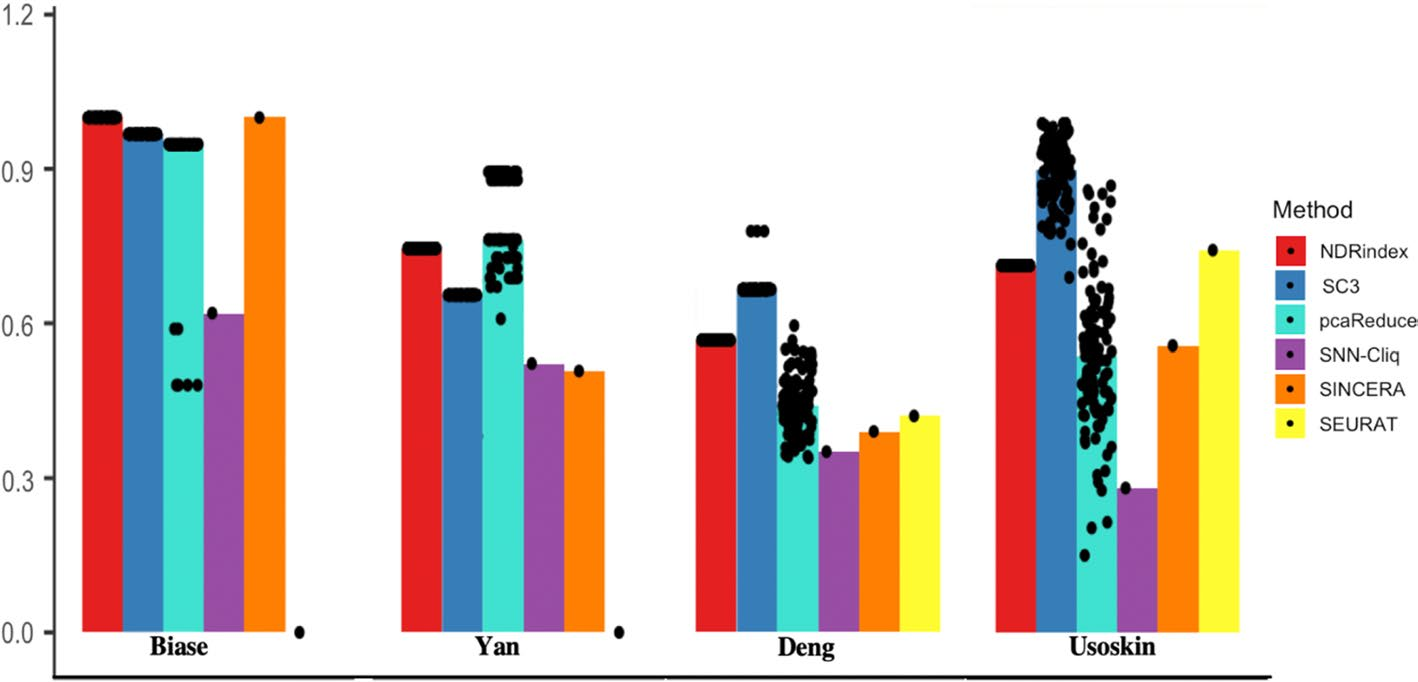
Comparison between NDRindex and other RNA-Seq processing methods. We submit the data that NDRindex choosen to hclust algorithm, and compare the result with other four methods (SC3, pcaReduce, SNN-Cliq, SINCERA, SRURAT) by comparing ARI. We run each method one hundred times, the dots represent the ARI between the inferred clusterings and reference labels of each running. and the height of rectangular represents the average ARI

To compare the performance of NDRindex, we applied the method to simulated and real data sets. The simulation dataset contains data of different quality. Some of them have obvious patterns and are suitable for grouping, while others are not. As shown in Fig. 3, the results show that our method can clearly distinguish them. For real datasets, we select five widely used single-cell RNA-Seq datasets, five normalization methods (TMM [6], Linnorm [11], scran [8], Seurat [18], scale)) and three dimension reduction methods (tSNE [15], PCA, sammon [14]). We collect the output of each combination of methods and subject them all to four typical clustering algorithms and compare the clustering results with ARI. As shown in Fig. 4, the result shows that the NDRindex algorithm chooses the data with the highest ARI, which shows that the NDRindex algorithm chooses a good combination of methods. We submit the data that NDRindex chosen to hierarchical clustering algorithm, and compare the result with other four methods (SC3 [5], pcaReduce [20], SNN-Cliq [21], SINCERA [22], SRURAT [18]) by ARI. As showed in Fig. 5, the performance of NDRindex shows its relatively high accuracy and stability.

## Discussion

For any REA-seq data, if there were at least one combination of normalization method and dimensionality reduction method, and the user believed that the optimal combination exists, NDRindex would be able to process as it is an evaluation to the best combinations of existing normalization methods and dimensionality reduction methods. If there is neither a defined normalization method nor dimensionality reduction, or the user cannot be sure whether at least one of the best combinations processes the data correctly, NDRindex would not be applicable. For instance, consider a data set consists of a homogeneous population of cells. If the user have multiple normalization methods and dimensionality reduction methods, NDRindex would be applicable. Since NDRindex is a method for evaluating combinations based on clustering trends and their results, it has no effect on the original data, so no new deviations will be introduced. The experiments shown by Figs. 4 and 5 have shown its accuracy, effectiveness, and bias are negligible.

## Conclusions

The computational analysis of single cell RNA-seq data is based on clustering models. The pre-processed data for normalization and dimensionality reduction have a significant impact on the results of the clustering.

In order to select a better combination of standardization and dimensionality reduction methods for preprocessing single-cell RNA-Seq data, we designed NDRindex to evaluate the data quality of preprocessing results by evaluating the clustering trend and degree of data aggregation. The result of both simulative data and the real data shows the effectiveness of NDRindex.

## Availability and requirements

- Project name: NDRindex.
- Project home page: https://github.com/zeromakerlovesmiku/NDRindex.
- Operating system(s): Platform independent.
- Programming language: R.
- Other requirements: R 3.4.4 or higher.
- License: GPL.

## Data Availability

NDRindex is available and open source at github (https://github.com/zeromakerlovesmiku/NDRindex), the datasets we used are listed in the references and are available.

## Abbreviations

RNAseq: RNA sequencing
scRNAseq: Single cell RNA sequencing
TMM: Trimmed mean of M-values
t-SNE: t-distributed stochastic neighbor embedding
